# Supplementary material for: Fluorescence Image Histology Pattern Transformation Using Image Style Transfer
Source: Front Oncol. 2019 Jun 25;9:519. doi: 10.3389/fonc.2019.00519 (PMC6603166; doi:10.3389/fonc.2019.00519)
Supplement: Supplementary file 1 [file Table_1.DOCX]

Supplementary Material

# Scoring protocol for quality assessment

| Score | Description |
| --- | --- |
| 0 | Negative* impact; Severe structures are removed |
| 1 | Negative impact; Moderate structures are removed |
| 2 | Negative impact; Slight structures are removed |
| 3 | No significant structures are removed |
| 4 | Positive** impact; Slight artifacts are removed |
| 5 | Positive impact; Moderate artifacts are removed |
| 6 | Positive impact; Severe artifacts are removed |

*** Negative impact** denotes that the transformed image has a lower diagnostic quality than the original image. (e.g. removing cells or other preexisting diagnostic features)

**** Positive impact** denotes that the transformed image has a higher diagnostic quality than the original image. (e.g. less artifacts)

| Score | Description |
| --- | --- |
| 0 | Negative* impact; Severe artifacts are added |
| 1 | Negative impact; Moderate artifacts are added |
| 2 | Negative impact; Slight artifacts are added |
| 3 | No significant structures are added |
| 4 | Positive** impact; Slight structures are added |
| 5 | Positive impact; Moderate structures are added |
| 6 | Positive impact; Severe structures are added |

*** Negative impact** denotes that the transformed image has a lower diagnostic quality than the original image. (e.g. hallucinating cells, misleading structures, and artifacts)

**** Positive impact** denotes that the transformed image has a higher diagnostic quality than the original image. (e.g. highlighting cells or other structures that were hard to notice)

# Style transferred CLE videos

Three temporal sequences of CLE images (from glioma tumors) were stylized. Each video shows the style transferred CLE frames alongside the original CLE images.
